# Antiviral Potential of Algae Polysaccharides Isolated from Marine Sources: A Review

**DOI:** 10.1155/2015/825203

**Published:** 2015-09-21

**Authors:** Azin Ahmadi, Soheil Zorofchian Moghadamtousi, Sazaly Abubakar, Keivan Zandi

**Affiliations:** ^1^Department of Medical Microbiology, Tropical Infectious Disease Research and Education Center (TIDREC), Faculty of Medicine, University of Malaya, 50603 Kuala Lumpur, Malaysia; ^2^Biochemistry Program, Institute of Biological Sciences, Faculty of Science, University of Malaya, 50603 Kuala Lumpur, Malaysia; ^3^The Persian Gulf Marine Biotechnology Research Center, Bushehr University of Medical Sciences, Bushehr 7514633341, Iran

## Abstract

From food to fertilizer, algal derived products are largely employed in assorted industries, including agricultural, biomedical, food, and pharmaceutical industries. Among different chemical compositions isolated from algae, polysaccharides are the most well-established compounds, which were subjected to a variety of studies due to extensive bioactivities. Over the past few decades, the promising results for antiviral potential of algae-derived polysaccharides have advocated them as inordinate candidates for pharmaceutical research. Numerous studies have isolated various algal polysaccharides possessing antiviral activities, including carrageenan, alginate, fucan, laminaran, and naviculan. In addition, different mechanisms of action have been reported for these polysaccharides, such as inhibiting the binding or internalization of virus into the host cells or suppressing DNA replication and protein synthesis. This review strives for compiling previous antiviral studies of algae-derived polysaccharides and their mechanism of action towards their development as natural antiviral agents for future investigations.

## 1. Introduction

Distinctive structure of viruses and their complicated life cycle have made the discovery of definite treatments against antiviral infections extremely demanding. Despite comprehensive studies for suitable vaccines and treatments against viral infections over the past half of a century, still several infections, such as human immunodeficiency virus (HIV), hepatitis C virus (HCV), and dengue virus (DENV), afflict a substantial proportion of the world populations in all generations [1–3]. Vaccine development against some viruses such as HIV and HCV was so far proved to be an intractable approach and there is no definite vaccine against numerous prevalent viral infections, including most respiratory-tract viruses, herpesviruses (HSV-1 and HSV-2), and human papilloma viruses (HPVs). Moreover, drug resistance to available antiviral agents by different viruses such as HIV type 1 (HIV-1) has always been a serious impediment to treatment of viral infections [4]. Until the 21st century, approximately less than only ten drugs were officially licensed against viral infections. Since then, better understanding of viral proliferation cycle and numerous researches have marked a quantum leap forward in the discovery of new antiviral drugs [5]. Nonetheless, despite some very real gains, we are still far more away from controlling the viral infections.

The unique living environment has gifted the marine world an assorted collection of algae from microorganisms to giant seaweeds. Various types of algae from microscopic diatoms to unicellular organisms and seaweeds reaching 30 m in length have stimulated significant economic interest as agar, fertilizer, food, source of iodine, and potash [6]. Amongst marine natural products, approximately 9% of biomedical compounds have been isolated from algae [7]. These marine organisms can synthetize assorted types of metabolisms, including polysaccharides, chlorophyll, acetogenins, fatty acids, vitamins, xanthophylls, amino acids, and halogenated compounds [8–11]. Despite being underexploited plant resources, recent investigations have established algae as a rich arsenal of active metabolites with pharmaceutical potential, including anticancer, antitumor, antioxidant, antiobesity, neuroprotective, antimicrobial, antinociceptive, anti-inflammatory, and antiangiogenic activities [8, 12–16].

## 2. Algae-Derived Polysaccharides 

Polysaccharides, also known as glycans, are the most abundant form of carbohydrate materials in the nature. Due to marked biological activities, numerous studies have been performed on different algae-derived polysaccharides, namely, agar, alginate, fucoidan, carrageenan, laminaran, proteoglycans, galactosyl glycerol, and rhamnan sulfate [9, 17–20]. The algae polysaccharides are natural polymers that are easily available in nature, nontoxic, cheap, safe, biodegradable, and biocompatible [21]. These polysaccharides have gained interesting and wide applications in the biomedical and pharmaceutical industries. Depending on the source of isolation, structure of some polysaccharides such as fucoidan can differ in the nature of constituents and the amount and length of their branching chains [22]. However, new synthetic routes are aiming to modify the biological activities of these polysaccharides through chemical modification and combination with other polymers [23].

## 3. Antiviral Activity of Algal Polysaccharides

A study by Gerber and colleagues in 1958 which showed inhibition of mumps and influenza B virus by polysaccharides from marine algae has introduced algae-derived polysaccharides as a potent source of antiviral agents [24]. Subsequently, antiviral activities of other polysaccharide fractions isolated from red algae have been reported against HSV and other viruses in the next two decades. Since then, numerous studies have published antiviral potential of various algae-derived polysaccharides and their underlying mechanism of action [25–28]. This review tries to summarize the antiviral activities of algae-derived polysaccharides and the mechanisms underlying these activities (see Table 1).

### 3.1. Carrageenan

**Figure 1:**
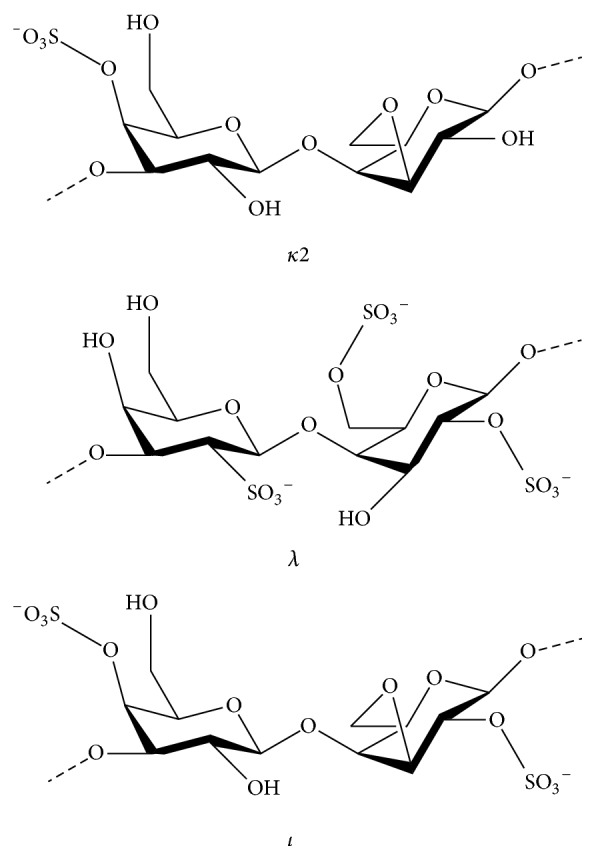
Chemical structure of carrageenan units, namely, kappa, lambda, and iota, isolated from red seaweeds.

Carrageenans are naturally occurring anionic sulfated polysaccharides (SPs), which appear as matrix material in great quantities by certain red algae (Rhodophyta), such as* Chondrus*,* Gigartina*,* Hypnea*, and* Eucheuma*, wherein they serve a structural function similarity to that of cellulose in plants [29]. The existence of 3,6-anhydrogalactopyranose and allocation of the sulfate groups on the main structures naturally classify carrageenan into three kinds, *λ*-, *κ*-, and *ι*-carrageenan (Figure 1) [30], and individually display special antiviral effects on several viral agents [31].

Carrageenans are selective inhibitors of several enveloped and nonenveloped viruses and act predominantly by inhibiting the binding or internalization of virus into the host cells [32, 33]. Carrageenans are exceptionally potent inhibitor of HPV* in vitro* by inhibiting the initial stage of infection [32]. Notably, they are also extremely effective against a range of sexually transmitted HPV types that lead to cervical cancer and genital warts [34, 35]. Carlucci and colleagues found that *λ*-type carrageenan is active against the replication of HSV upon its firm interaction that leads to inactivation of HSV virion [36]. They also discovered that the *λ*-carrageenan and moderately cyclized *μ*/*ι*-carrageenan isolated from* Gigartina skottsbergii* exert promising antiviral activities towards diverse strains of HSV-1 and HSV-2 during virus attachment stage [37, 38]. Surprisingly, similar results were reported by different group of researchers, who analysed the chemical structure and antiviral activity of carrageenan (*lambda*,* kappa*, and* iota*) against HSV-2 infection [39, 40]. A recent* in vitro* study conducted by Grassauer and colleagues reported the inhibitory effects of *ι*-carrageenan against human rhinovirus (HRV) proliferation by preventing the primary phases of virus replication. They have suggested that this effect is possibly attributed to the suppression of the allosteric activity of virus particles during their entry [33]. Additionally, *ι*-carrageenan was proven to be effective against dengue virus replication in mosquito and mammalian cells; however, the mode of antiviral action of *ι*-carrageenan in both cell types was interestingly distinctive. In Vero cell line, the inhibitory activity has been exerted at early stage of virus adhesion probably due to some primary receptors, whereas in mosquito cell it affected the cell proliferation and protein synthesis [41, 42].

A recent* in vivo *study in mice has revealed that the low molecular weight carrageenans (3, 5, and 10 kDa), as well as acetylated and sulfated derivatives, have substantial inhibitory effects against influenza virus. Furthermore, the smallest *κ*-carrageenan with appropriate sulfation and acetylation degree was the greatest antiviral candidate against influenza virus* in vivo* [43]. Yamada and colleagues remarked the fact that the antiviral activities of carrageenans are extremely correlated with their molecular weights and existence of sulfation groups, since different molecular weight* O*-acylated carrageenans reduced the HIV activity by depolymerisation and sulfation process [44].

### 3.2. Galactan

**Figure 2:**
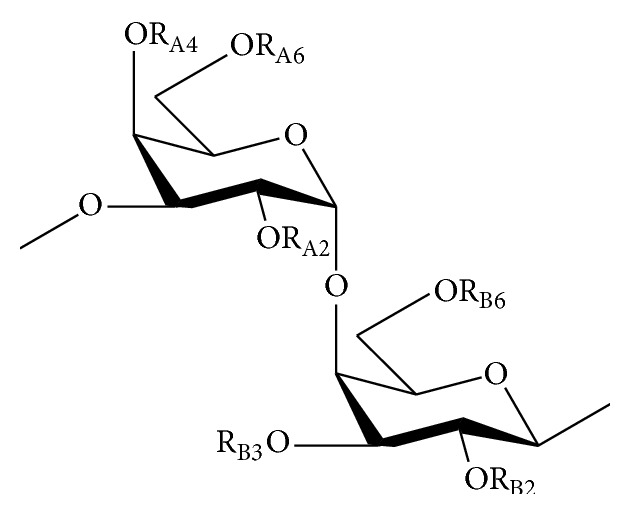
Chemical structure of galactan units isolated from red algae. R_A2_: SO_3_^−^, H; R_A4_: SO_3_^−^, H, pyruvic acid; R_A6_: SO_3_^−^, H, CH_3_, pyruvic acid; R_B2_: SO_3_^−^, H, CH_3_; R_B3_: H; R_B6_: SO_3_^−^, H.

The main extracellular polysaccharides of red algae are known as sulfated galactans. They are made up of linear chains of galactoses with a few exceptions; a chain of alternating 3-*β*-D-galactopyranose (G units) and 4-*α*-D-galactopyranose residues or 4-3,6-anhydrogalactopyranose residues complete their structural backbone with presence of D-series (D unit) in carrageenans and L-series (L unit) in agarans (Figure 2) [45, 46]. Another exceptional collection of galactans also exists: the DL-hybrids that enclose G unit attached to both D and L units [47, 48].

The various structural types of these polysaccharides have shown a vigorous antiviral potency against several enveloped viruses, such as HSV-1 and HSV-2, DENV, HIV-1 and HIV-2, and hepatitis A virus [49, 50]. Three galactan polysaccharide fractions from the Argentinian marine alga* Callophyllis variegata* were isolated and purified by Rodríguez and colleagues in 2005. In addition to the structural illustration of main fractions, the antiviral effect of these fractions was assessed against HSV-1 and HSV-2 and DENV-2, which surprisingly exhibited potent inhibitory effects with low cytotoxicity together. Therefore, it was suggested that these compounds might develop “promising antiviral agents” [51]. It was also reported that galactan sulfate (GS), isolated from* Agardhiella tenera*, displays an effective control against HIV-1 and HIV-2. GS blocked the adhesion of virus to cell, in addition to the attachment of gp120 on CD4+ T cell receptor to HIV-1 gp120. Consistently, GS at the concentration of >5 g/L blocked syncytia emergence in Molt-4 cells and HIV-1 or HIV-2 infected HUT-78 cells [49]. Matsuhiro and Colleagues discovered the antiviral property of a sulfated galactan isolated from the marine red seaweed* Schizymenia binderi* by reviewing its structural configuration. This sulfated galactan clearly presented highly selective antiviral effect against HSV types 1 and 2 with lowest cytotoxicity. Its inhibitory effects are suggested to be involved in the attachment of virus to host cells [52]. Moreover, Talarico and colleagues examined the antiviral activity of a D,L-galactan hybrid C2S-3, extracted from the Brazilian marine alga* Cryptonemia crenulata*, in the multiplication of DENV-2 in Vero cell line. As this compound lacked any cytotoxicity to Vero cell line, it is indicated that it is effective against three clinical strains of DENV-2 (IC_50_ = 0.8–16 *μ*g/mL). Further mechanistic work concluded that the compound affects the initial steps of virus adsorption and entry into the host cells; therefore C2S-3 is supported to be a “promising DENV-2 antientry” as well [53].

### 3.3. Alginate

**Figure 3:**
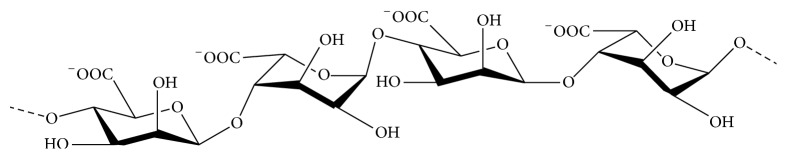
Chemical structure of alginate polysaccharide (GM blocks).

Alginates are the principle cell wall acidic polysaccharides widely distributed in brown algae (Phaeophyceae) including* Laminaria hyperborea*,* Laminaria digitata*,* Laminaria japonica*,* Ascophyllum nodosum*, and* Macrocystis pyrifera*. They are mostly extracted from bubble Zosteraceae, kelp, macro algae, and marjoram algae, respectively. Alginates are linear anionic polysaccharides, composed of a main backbone of poly-D-glucuronic acid (G blocks) and poly-D-mannuronic acid (M blocks), together with D-guluronic acid and D-mannuronic acid (GM blocks) usually alternating (Figure 3) [54, 55]. Alginates have found numerous applications in biomedical science and engineering and have been particularly attractive for their antiviral activities.

**Figure 4:**
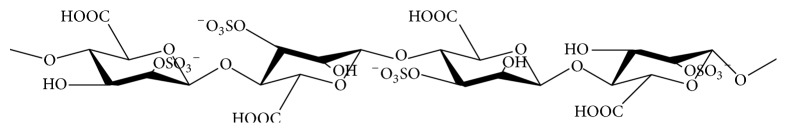
Chemical structure of sulfated polymannuroguluronate (SPMG).

A prominent marine polysaccharide drug named 911 derived from alginate polysaccharide exhibited promising activity against HIV-1 at both chronic infection of H9 cells and acute infection of MT4 cells* in vitro* and* in vivo*. These special effects revealed that 911 drug inhibited the viral replication of HIV via significantly decrementing the activity of reverse transcriptase (RTase), discontinuing the virus adsorption, and improving the defense mechanisms of the host cells [56, 57]. Alternative inhibitory result was also reported later for hepatitis B virus (HBV) that 911 drug could inhibit the virus replication by suppressing the activity of DNA polymerase activity [58]. Wang and colleagues discovered that the sulfated polymannuroguluronate (SPMG) (Figure 4) [55], the sulfated form of alginate, is a characteristic anti-AIDS drug candidate, as it caused the inhibition of HIV-1 infection mainly through the robust attachment of virus gp120 protein with CD4 molecules on the surface of T cells. Moreover, there is huge correlation between the size of SPMG oligosaccharides and their inhibitory significance that the octasaccharide will be the minimal active fragment preventing syncytium formation and reducing the P24 core antigen level in HIV-IIIB-infected CEM cells [59, 60].

### 3.4. Fucan and Fucoidan

Fucans are high molecular weight sulfated polysaccharides, usually classified into three major groups: glycuronogalactofucans, fucoidans, and xylofucoglycuronans. These polymers occur in the intercellular tissues or mucilaginous matrix of brown algae. They are widely distributed in the cell walls of brown algae and regarded only as a huge source of L-fucose with different portions of neutral sugars such as galactose, glucose, mannose, and uronic acid that can occur in the polymer [61]. Fucose is attached to the central backbone, mainly bound by 1 → 2 glycosidic linkages, forming branching points at every 2-3 fucose residues within the chain [62]. The structure of algal fucans varies among species and sometimes among different kinds of the seaweed [63, 64]. Fucans have a broad spectrum of biological activities. However, the structure of algal fucans varies among species and sometimes among different parts of the seaweed [63, 64]. Thus, each new purified sulfated fucan is a unique compound and thus a potential new drug.

Beside many other well-attested responsibilities, such antiproliferative, antiadhesive effects on cells can especially protect the cells from viral infections [65]. Queiroz and colleagues evidence that the sulfated fucans from such seaweed species* Dictyota mertensii*,* Lobophora variegata*,* Fucus vesiculosus*, and* Spatoglossum schroederi* could prevent HIV infection via blocking the activity of reverse transcriptase and notably their results strongly indicated the necessity of sulfate and carboxyl group in the inhibitory activity of these polysaccharides [66]. A fucan polysaccharide isolated from* Cladosiphon okamuranus* with glucuronic acid and sulfated fucose units' composition inhibited DENV-2 infection in BHK-21 cell line. Conversely, less effect was observed on three other serotypes of DENV (DENV-1, DENV-3, and DENV-4) [67]. Further investigation of the envelope glycoprotein compositions from the existing four serotypes of DENV revealed that arginine-323 in DENV-2 plays an important role upon its interaction with the fucan and content of sulfation of fucan was also vital for this activity [45]. Akamatsu and colleagues evaluated an effective anti-influenza virus compound named MC26, a new type of fucose polysaccharides isolated from marine brown algae species,* Sargassum piluliferum*. It exhibited a stronger anti-influenza virus activity with low cytotoxicity* in vivo* and* in vitro* as compared with known active compounds [68]. Furthermore, Mandal and colleagues studied the sulfated fucans extracted from brown seaweed* Cystoseira indica*, which exhibited a promising activity against HSV-1 and HSV-2 deprived of any cytotoxicity for Vero cell cultures; however no direct inactivating effect on virions in a virucidal assay was detected. Hence, it was suggested that the mode of action of these compounds could be possibly due to the inhibition of virus adsorption [69].

**Figure 5:**
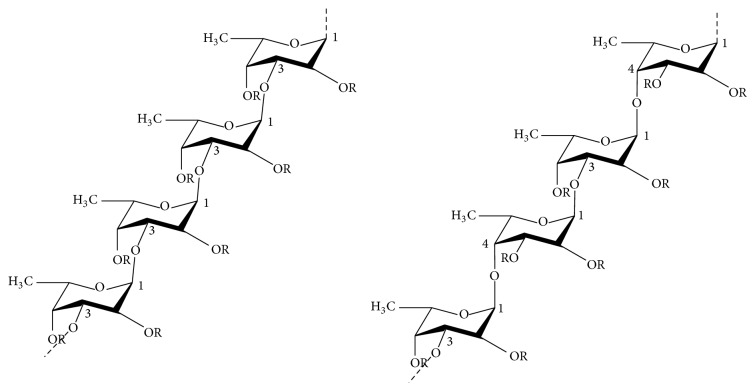
Chemical structures of two different backbones for fucoidan. R groups demonstrate potential places for attachment of carbohydrate (*α*-L-fucopyranose and *α*-D-glucuronic acid) and noncarbohydrate (sulfate and acetyl groups) substituents.

Fucoidan is a term used to define a polysaccharide based mainly on sulfated L-fucose and less than 10% on other monosaccharides. The term sulfated fucan can be used to define heterofucans containing sulfated fucose and neutral sugars. However, fucans and fucoidans are often used interchangeably. Fucoidan has a high proportion of fucose in the extracellular matrix of several brown algae such as mozuku, komby, limu moui, bladderwrack, wakame, hijiki, and sea cucumber. The main skeleton of fucoidans involves a-1,3-linked sulfated L-fucose, a repeating sequence of alternating a-(1-3)- with the possible a-(1-4)-glycosidic bonds. The chemical structure and composition of fucoidans are considerably diverse, depending highly on the isolated species, which usually are sulfated and acetylated and may also hold uronic acid (Figure 5) [70–72].

Fucoidan possesses various biological activities such as activity against many RNA and DNA viruses both* in vivo* and* in vitro*, including important human pathogens such as HIV, HSV1-2, dengue virus, and cytomegalovirus [67, 73]. Fucoidans demonstrated their antiviral activities by mainly blocking the interaction of viruses to the cells so as to inhibit viral-induced syncytium formation [74]. Isolated fucoidans from several species,* Adenocytis utricularis* [75],* Undaria pinnatifida* (Mekabu) [76],* Stoechospermum marginatum* [77],* Undaria pinnatifida* [78, 79], and* Cystoseira indica* [69], exhibited potential antiviral effects against HSV-1 and HSV-2 deprived of cytotoxicity for Vero cell cultures. Elizondo-Gonzalez and colleagues reported that the isolated fucoidan from* Cladosiphon okamuranus *showed potent antiviral activity against Newcastle disease virus in the Vero cell line at the initial stages of infection. The viral-induced-syncytial formation declined by exposure of fucoidan prior to cleavage of the fusion protein, which led to attachment of fucoidan to the F0 protein. Consequently, fucoidan exhibited a better antiviral potency than ribavirin [80]. Moen and Clark, 1993, studied isolated fucoidan from* F. vesiculosus*, which showed its potential to suppress HIV RT* in vitro*. Unexpectedly, preincubation of cell-free virus to 200 mg/mL caused 100% diminution in the amount of HIV-1 p24 antigen release. These studies showed that respective activities are not because of dispatching of target cells. Indeed, fucoidan induced no adverse effects on protein metabolism and cell generation. HIV-1 infection of target cells is actually protected after preincubation with fucoidan. Moreover, fucoidan can effectively augment immune system health by activating immunoreactions of the cellular and humoral types and by increasing macrophage phagocytosis [62, 81]. In conclusion, fucoidan directly affects the secretion of extracellular matrix proteins, influences the proliferation of cells, and can activate apoptosis [82–85].

### 3.5. Laminaran

**Figure 6:**
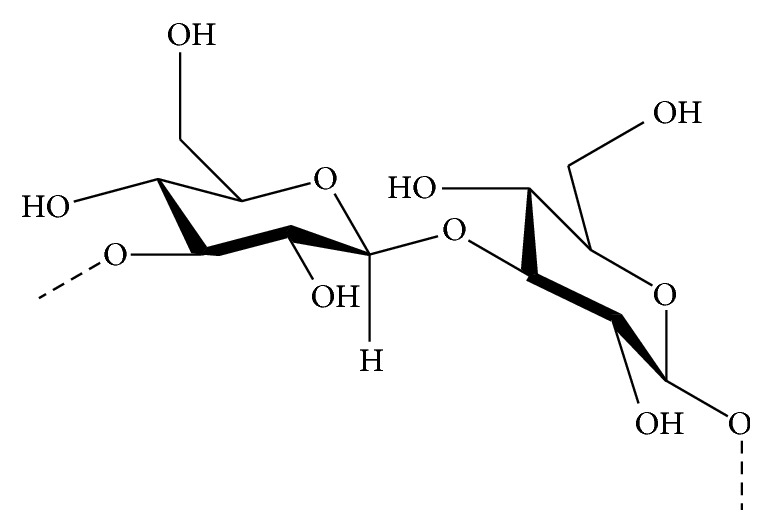
Chemical structure of glucose in laminaran.

Laminaran, a glucan, is one of the common polysaccharides abundant in a wide variety of brown algae such as* F. vesiculosus*,* Saccharina longicruris*, and* Ascophyllum nodosum*. It is a linear polysaccharide made up of *β*-(1 → 3)-linked glucose in the central chain, with *β*-(1 → 6)-linked side-chain branching (Figure 6) [30, 86]. Thus far, two kinds of laminaran are recognized: one kind is made of glucose residues (G-series), whereas the other kind is terminated by D-mannitol residues (M-series) [87]. Therefore, the ratios of the two kinds of laminaran, together with their structural configurations, are changeable according to the isolated species, as well as environmental factors predicted to directly affect the biological properties of laminaran [88, 89]. Laminaran is created by photosynthesis and exhibits a great antiviral activity and low toxicity* in vivo *[90]. Muto and colleagues reported that laminaran polysaccharides extracted from kelp are proficient to prevent the activity of HIV by preventing the adsorption of HIV on human-derived lymphocytes and the ability of HIV reverse transcriptase, which play an important role for the virus proliferation. This study suggested that laminaran polysaccharides are effective inhibitors on HIV replication and proliferation [91].

### 3.6. Naviculan

Naviculan is a sulfated polysaccharide from a diatom called* Navicula directa*. This compound is made of several sugars such galactose, xylose, rhamnose, fucose mannose, and sulphate with a high molecular weight. Lee and colleagues reported that naviculan has a potent antiviral activity against HSV-1 and HSV-2 (IC_50_: 7–14 *μ*g/mL) and influenza virus by inhibiting the initial stages of viral replication, possibly blocking viral internalization into host cells. Moreover, naviculan displayed a notable inhibitory effect on fusion between the cells that express CD4 receptor and HIV gp160-expressing HeLa cell line, which was used as a model system of HIV infection. This study proposed naviculan as a novel antiviral sulfated polysaccharide with a wide range of activities against enveloped viruses [92].

### 3.7. p-KG03

The marine microalga* Gyrodinium impudicum* strain KG03 produces a highly sulfated exopolysaccharide p-KG03. The p-KG03 polysaccharide is unique compound with the molecular weight of 1.87 × 10^7^, categorized as a homopolysaccharide of galactose conjugated with uronic acid and sulfate groups. P-KG03 was the first reported marine compound to impressively suppress tumor cell growth and infection by encephalomyocarditis virus (EMCV)* in vitro* (EC_50_ = 26.9 *μ*g/mL). It was shown that the development of cytopathic effects in HeLa cells infected by EMCV was either suppressed entirely or reduced by differing the p-KG03 concentration [93]. Moreover, Kim and colleagues reported that the p-KG03 exhibits great inhibitory activity on influenza A virus infection but not all influenza B viruses* in vitro*. This study highlighted that the virus replication was declined when p-KG03 was added throughout the infection activity, meaning that these compounds mainly target the viral adsorption and internalization steps. Accordingly, the polysaccharide p-KG03 not only is capable of preventing the attachment of influenza virus to host cells but also blocks the cellular internalization of the virus and early stages of replication. Hence, the potential activities of p-KG03 evidenced that the sulfated metabolites from marine systems could be a possible candidate for drug development [94].

### 3.8. A1 and A2

Extracellular sulfated polysaccharides A1 and A2 are found inmarine microalga,* Cochlodinium polykrikoides*. These polysaccharides are constituted of glucose, galactose, mannose, and uronic acid, with distribution of sulfate groups. The cytopathogenic effects of HIV- 1 in MT-4 cells, influenza virus types A and B in MDCK cells, and respiratory syncytial virus types A and B in Hep-2 cells were inhibited by A1 and A2. It was approved that A1 polysaccharides are also effective against HSV-1, whilst A2 polysaccharides are effective against parainfluenza virus type 2, both in HMV-2 cell line. Both A1 and A2 polysaccharides are noncytotoxic at 100 *μ*g/mL and generate weak (10%) inhibitory effects on blood coagulation at specific concentrations that cause viral inhibition [95].

### 3.9. Calcium Spirulan

A novel sulfated polysaccharide, termed calcium spirulan (Ca-SP), was isolated from a marine blue-green alga,* Arthrospira platensis* (previously called* Spirulina platensis*). This polysaccharide is found to be composed of mannose, ribose, fructose, glucose, xylose, galactose, rhamnose, galacturonic acid, glucuronic acid, calcium, and sulfate. Hayashi and colleagues in 1996 found Ca-SP to be the selective inhibitor of various viruses, including HSV-1 (in HeLa cells), HCMV (in HEL cells), influenza A (in MDCK cells), Coxsackie virus (in Vero cells), measles (in Vero cells), HIV-1 (in MT-4 cells), polio (in Vero cells), and mumps (in Vero cells). This study revealed that the antiviral activities of Ca-SP are attributed to inhibition of virus entry into host cells and Ca-SP was also shown to have a small anticoagulant activity [96]. Lastly, Hayashi and colleagues expanded their discoveries by comparing the anti-HIV-1 and anti-HSP-1 activities of Ca-SP with antiviral effects of dextran sulfate (DS) as a symbolic sulfated polysaccharide. Their results indicated that Ca-SP is an applicable antiviral compound against both HSV-1 and HIV-1. Therefore, Ca-SP became known as a relatively promising anti-HIV polysaccharide since, at low concentrations of Ca-SP, formation of virus-induced syncytium did not happen. More importantly, presence of calcium was found to be pivotal for dose-dependent suppression of syncytium formation and cytopathic effect induced by HIV-1. In conclusion, by overcoming the drawbacks reported for other sulfated polysaccharides, Ca-SP can be a promising candidate for development of new anti-HIV drugs [96].

### 3.10. Nostoflan

Nostoflan (NSF) is an acidic polysaccharide found in edible blue-green alga,* Nostoc flagelliforme.* The structural analyses of NSF revealed that it is mainly made of sugar sequences of (→4)-*β*-d-Glc*p*-(1 → 4)-d-Xyl*p*-(1 and →4)-[*β*-d-GlcA*p*-(1 → 6)-]-*β*-d-Glc*p*-(1 → 4)-d-Gal*p*-(1→). Kanekiyo and colleagues identified that nostoflan has a noble inhibitory effect on several enveloped viruses including HSV-1 and HSV-2, human cytomegalovirus, and influenza A virus. The most sensitive stage of viral replication to nostoflan in time of addition during experiment was assumed to be the initial stage of infection covering the virus binding or/and internalization processes. But further investigation confirmed that uniquely the inhibition of virus binding to host cells was responsible for the antiherpetic effect induced by nostoflan and their findings recommended nostoflan as a great antiherpes candidate [97].

### 3.11. Sea Algae Extract

Sea algae extract (SAE) is a sulfated polysaccharide with the high molecular weight isolated from marine red alga* Schizymenia pacifica*. SAE is a member of *λ*-carrageenan, which is composed of galactose (73%), sulfonate (20%), and 3,6-anhydrogalactose (0.65%), and it is known that SEA is a selective inhibitor of HIV reverse transcriptase and replication* in vitro* and has no adverse effect on cell proliferation [98]. Similar results are obtained once a cell-free system was employed to examine the effect of SEA on reverse transcriptase from avian retrovirus (avian myeloblastosis virus) and mammalian retrovirus (Rauscher murine leukemia virus). The SEA exhibited inhibitory effects on reverse transcriptase of both retroviruses but elicited no effects on the function of cellular DNA polymerase alpha and RNA polymerase II* in vitro*. Accordingly, it is unlikely to have an adverse effect on the proliferation of cell culture. It is hypothesized that sulfate residues play a vital role in RT suppression activity. This hypothesis is substantiated by inducing activity in polysaccharides [99].

## 4. Conclusions 

A variety of biological activities are reported for algal polysaccharides including the transcendent antiviral effect. In the majority of cases, the antiviral activity of these polysaccharides is exerted through suppression of virus adhesion to the host cells. Throughout our literature review, it was noticed that despite numerous investigations which substantiated the antiviral potential of algal polysaccharides, few aspects may have been neglected by researchers to date. Many of the reported pharmacological studies were only limited to* in vitro* investigation in a single host cells. However,* in vivo* examination is pivotal for the development of pharmaceutical drugs. In addition, further studies including clinical trials to exploit the antiviral activity against viral infections in human are another area for further research. To the best of our knowledge, the synergistic activity of algal polysaccharides against viral infections was not adequately covered in previous studies. It is hoped that this review would be a source of enlightenment and motivation for the interested researchers to conduct further* in vitro*,* in vivo*, and clinical analyses on the antiviral activity of algal polysaccharides with a view to developing new antiviral drugs.

## Figures and Tables

**Table 1:** Antiviral activities of algae polysaccharides derived from marine sources.

Antiviral polysaccharide	Organism	Virus
Carrageenan	Red alga*, Gigartina skottsbergii *	Influenza virus, DENV, HSV-1, HSV-2, HPV, HRV, HIV

Galactan	Red algae, *Callophyllis variegate, Agardhiella tenera, Schizymenia binderi, Cryptonemia crenulata *	HSV-1, HSV-2, HIV-1, HIV-2, DENV, HAV

Alginate	Brown algae, *Laminaria hyperborea*, *Laminaria digitata*, *Laminaria japonica*, *Ascophyllum nodosum*, *Macrocystis pyrifera *	HIV, IAV, HBV

Fucan	Brown algae, *Adenocytis utricularis*, *Undaria pinnatifida*, *Stoechospermum marginatum*, *Cystoseira indica*, *Cladosiphon okamuranus*, *Fucus vesiculosus *	HSV-1, HSV-2, HCMV, VSV, Sindbis virus, HIV-1

Laminaran	Brown algae, *Fucus vesiculosus*, *Saccharina longicruris*, *Ascophyllum nodosum *	HIV* *

Naviculan	Diatom, *Navicula directa *	HSV-1, HSV-2

p-KG03	Microalga, *Gyrodinium impudicum *	EMCV, influenza A virus

A1 and A2	Microalga, *Cochlodinium polykrikoides *	Influenza A and B viruses, RSV-A, RSV-B, parainfluenza-2

Calcium spirulan	Blue-green alga, *Arthrospira platensis *	HSV-1, measles, mumps, influenza, polio, Coxsackie, HIV-1, HCMV

Nostaflan	Blue-green alga, *Nostoc flagelliforme *	HSV-1, HSV-2, influenza A virus, human cytomegalovirus

Sea algae extract	Red alga, *Schizymenia pacifica *	HIV, AMV, RMLV
